# Conceptual Comparison of Population Based Metaheuristics for Engineering Problems

**DOI:** 10.1155/2015/936106

**Published:** 2015-03-19

**Authors:** Oluwole Adekanmbi, Paul Green

**Affiliations:** Department of Finance and Information Management, Durban University of Technology, P.O. Box 101112, Scottsville, Pietermaritzburg 3209, South Africa

## Abstract

Metaheuristic algorithms are well-known optimization tools which have been employed for solving a wide range of optimization problems. Several extensions of differential evolution have been adopted in solving constrained and nonconstrained multiobjective optimization problems, but in this study, the third version of generalized differential evolution (GDE) is used for solving practical engineering problems. GDE3 metaheuristic modifies the selection process of the basic differential evolution and extends DE/rand/1/bin strategy in solving practical applications. The performance of the metaheuristic is investigated through engineering design optimization problems and the results are reported. The comparison of the numerical results with those of other metaheuristic techniques demonstrates the promising performance of the algorithm as a robust optimization tool for practical purposes.

## 1. Introduction

In structural engineering, most design optimization problems are highly nonlinear consisting of different design variables and complex constraints such as displacements, geometrical configuration, stresses, and load carrying capability. The design variables are normally grouped into two categories, namely, continuous variables and discrete variables. Optimization problems involving continuous and discrete variables generally require problem-specific search techniques [1]. Evolutionary multiobjective optimization techniques are examples of problem-specific search techniques. Several literatures have applied evolutionary multiobjective optimization techniques to solving multiobjective optimization problems to find a set of trade-off optimal solutions. Since most engineering problems involve multiobjective optimization, it is appropriate to apply an evolutionary optimization algorithm to solve them.

In the last two decades, different types of techniques aimed at effectively and efficiently exploring a search space by combining several basic heuristic methods have emerged [2–4]. These techniques currently referred to as “*Metaheuristics*” are used to describe heuristic methods applied to solving different practical problems.* Metaheuristics* can be considered as a global algorithmic framework used in solving several optimization problems with little changes, thereby making the algorithm adaptive to the specific problem [5].

Metaheuristic search techniques, such as simulated annealing (SA) [6], genetic algorithm (GA) [7], evolution strategies (ESs) [8], and particle swarm optimization (PSO) [9], which are generally developed based on natural phenomena have become the popular optimization techniques of recent years due to their capability of finding promising solutions for complicated optimization problems as well as their independence to the derivatives of objective functions.

Furthermore, metaheuristics can handle both discrete and real-valued variables and can be applied to a wide range of optimization problems effectively. Basically, both trajectory and population based metaheuristic approaches aim to locate the global optimum in the solution space through random moves. The key difference between the metaheuristics is in the way they propose the next move in the solution space. This motivates developers of optimization algorithms to find more efficient methodologies for originating robust optimization algorithms. However, sometimes this results in complicated approaches which are difficult to understand and implement. Hence, this study is an attempt to test the simplicity and efficiency methodology of GDE3 metaheuristics in solving engineering optimization purposes.  Section 2 describes the GDE3 metaheuristics briefly. Test cases are described and optimization results are discussed in Section 3.  Section 4 provides a clear conclusion of the study.

## 2. Generalized Differential Evolution Metaheuristic

Several extensions of differential evolution [26] exist for solving constrained and nonconstrained multiobjective optimization problems [27, 28]. In comparison to the extension of differential evolution (DE), GDE3 makes differential evolution a suitable algorithm for multiobjective optimization as well as constrained optimization with little changes to the basic differential evolution algorithm. GDE3 extends DE/rand/1/bin strategy which exhibit slow convergence rates and strong exploration properties. GDE3 is a third version of generalized differential evolution modifying the selection process of the basic differential evolution algorithm [29]. The selection process in GDE3 is guided by these three rules:

- In a scenario where both the old vector and trial vector are infeasible, the old vector is selected if it dominates the trial vector, but if the trial vector weakly dominates the old vector, then the trial vector is selected.
- Feasible vector is selected in a situation where both feasible and infeasible vectors are generated.
- In a scenario where both the old vector and trial vector are feasible, the old vector is selected if it dominates the trial vector, but if the trial vector weakly dominates the old vector, then the trial vector is selected.

The whole GDE3 is presented in Algorithm 1. Parts that are new compared to previous GDE versions are framed in Algorithm 1. Without these parts, the algorithm is identical to GDE1. GDE3 can be seen as a combination of GDE2 and Pareto Differential Evolution Approach (PDEA). GDE3 is similar to differential evolution for multiobjective optimization (DEMO) except that DEMO does not contain constraint handling nor recede to basic DE in the case of a single objective because DEMO modifies the basic DE and does not consider weak dominance in the selection. Moreover, GDE3 has an improved diversity maintenance compared to DEMO. There are no constraints to be evaluated when *K* = 0 and *M* = 1, and the selection is simply

(1) $$\begin{array}{c} x_{i,G+1}=\left\{\begin{array}{cc} u_{i,G}, & \text{if}\;\;f\left(u_{i,G}\right)\leq f\left(x_{i,G}\right),\\ x_{i,G}, & \text{otherwise}. \end{array}\right. \end{array}$$

This is the same as for the basic DE algorithm. The size of the population does not increase since this requires that *x*_*i*,*G*_ and *u*_*i*,*G*_ do not dominate each other even weakly, but in the case of a single objective, the reverse is the case. GDE3 performs the sorting of the vector by calculating the crowding distance of the vector. The selection process based on crowding distance gives GDE3 an advantage over NSGAII. In the case of comparing feasible, incomparable, and nondominating solutions, both offspring and parent vectors are saved for the population of the next generation [4]. There is no need to remove elements, since the population size does not increase. Hence, GDE3 is identical to basic DE in this case. GDE3 improves the ability to handle multiobjective optimization problems by giving a better distributed set of solutions and are less sensitive to the selection of control parameter values compared to the earlier GDE versions. As a result, this procedure reduces the computational costs of the metaheuristic and improves its efficiency. Readers interested in GDE3 should refer to the texts by [30, 31].

## 3. Implementation of Engineering Optimization Problems

The metaheuristic optimization was implemented in NETBEAN v7.3; optimization runs were executed on an HP PC with a 2.30 GHz Intel Dual Core processor and 4 GB of RAM memory. Different examples taken from several optimization literatures were used to show the performance of GDE3 metaheuristic. These examples have been previously solved using a variety of other techniques, which is useful to show the validity and effectiveness of the GDE3 metaheuristic. The optimal results were compared with data recently published in literatures. An experiment has been performed to determine the best values of *F* and CR for better performance in GDE3 metaheuristic. For this purpose, both CR and *F* are varied from 0.1 to 1 with an increment of 0.1. The simulations were conducted for each value of *F* with respect to all values of CR. Hence, 100 such simulations were conducted. From the results, it was found that better Pareto optimal front is obtained by GDE3 with *F* = 0.5, CR = 0.9 and the termination condition is set to the 10,000 objective function evaluations.

**Example 1 (welded beam design optimization problem).**

**Figure 1:**
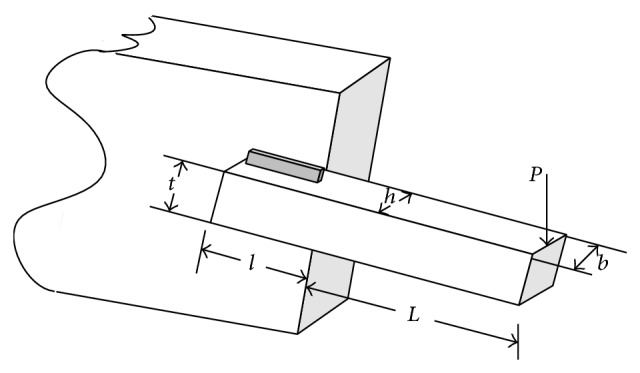
Schematic of the welded beam design problem [1].

The welded beam problem is designed to minimize the fabrication cost by subjecting it to some constraints such as bending stress (*σ*), shear stress (*τ*), end deflection (*δ*), and buckling load (*P*_*c*_). The design variables of the optimization problem are the thickness of the beam (*b*), the thickness of the weld (*h*), the welded joint length (*l*), and the beam width (*t*).  Figure 1 shows the welded beam design structure.

The values of *l* and *h* must be integer multiples of 0.0065 in. Assuming *x*_1_ =* h*, *x*_2_ =* l*, *x*_3_ =* t, *and *x*_4_ =* b *as design variables, the optimization problem can be mathematically expressed as follows:

(2) Minimize f(x→)=(1+C1)x12x2         +C2x3x414.0+x2,Subject to g1x→=τx→−τmax⁡≤0,      g2(x→)=σ(x→)−σmax⁡≤0,      g3x→=x1−x4≤0,     g4x→=C1x12         +C2x3x414.0+x2−5.0≤0,      g5(x→)=0.125−x1≤0,      g6(x→)=δ(x→)−δmax⁡≤0,      g7(x→)=P−Pc≤0,

where

(3) $$\begin{array}{c} \tau \left(\vec{x}\right)=\sqrt{{\left(\tau^{\prime }\right)}^{2}+\left(2\tau^{\prime }\tau^{\prime \prime }\right)\frac{x_{2}}{2R}+{\left(\tau^{\prime \prime }\right)}^{2}},\\ \tau^{\prime \prime }=\frac{MR}{J},\\ M=P\left(L+\frac{x_{2}}{2}\right),\\ R=\sqrt{\frac{{x_{2}}^{2}}{4}+{\left(\frac{x_{1}+x_{3}}{2}\right)}^{2}},\\ J=2\left\{\sqrt{2}x_{1}x_{2}\left[\frac{{x_{2}}^{2}}{12}+{\left(\frac{x_{1}+x_{3}}{2}\right)}^{2}\right]\right\},\\ \sigma \left(\vec{x}\right)=\frac{6PL}{x_{4}{x_{3}}^{2}},\\ \tau^{\prime }=\frac{P}{\sqrt{2}x_{1}x_{2}},\\ \delta \left(\vec{x}\right)=\frac{4PL^{3}}{E{x_{3}}^{3}x_{4}},\\ P_{c}\left(\vec{x}\right)=\frac{4.013E\sqrt{{x_{3}}^{2}{x_{4}}^{6}/36}}{L^{2}}\left(1-\frac{x_{3}}{2L}\sqrt{\frac{E}{4G}}\right). \end{array}$$

The simple bounds of the problem are *x*_1_, *x*_4_ ∈ [0.1, 2.0] and *x*_2_, *x*_3_ ∈ [0.1, 10.0]. The values of parameters involved in the formulation of the welded beam problem are also shown in Table 1.

The optimum design of the welded beam is executed using GDE3 metaheuristic, and the best solution is found as *x* ^*^ = {*x*_1_,  *x*_2_,  *x*_3_,  *x*_4_} = {0.20572840999876, 3.47072911158159, 9.03661683005891, 0.20572540074781} which yields an objective function value of $f(\vec{x})$ = 1.7248496 as seen in Table 2.

The results obtained by GDE3 are presented in Table 2. GDE3 found the global optimum requiring 400 iterations (i.e., 10,000 evaluations) per optimization run.  Table 3 provides a comparison of this solution with the results of other optimization algorithms. It is apparent from the table that GDE3 metaheuristic finds a competitive solution using only 10,000 evaluations which is considerably lesser than those of other approaches. Further, a statistical evaluation of 100 independent runs of the GDE3 metaheuristic is tabulated in Table 4 considering the best, worst, average, and the standard deviation (std. dev.) of the obtained solutions. The ratio between the optimized costs corresponding to best and worst designs is 1.00042. Remarkably, GDE3 produced the overall best design result with a value of 1.724849. For continuous optimization problem, [20, 22] found a better design result with a value of 1.7248 at a higher function evaluation.

**Example 2 (pressure vessel optimization problem).**

The pressure vessel problem is designed to minimize total cost which is comprised of the welding cost and forming material cost. The compressed air tank with a working pressure of 3000 psi and a minimum volume of 750 ft^3^ must be designed according to the ASME code on boilers and pressure vessels. The design variables of the optimization problem are the length of the cylindrical segment of the vessel (*L*), the thickness of the cylindrical skin (*T*_*s*_), the inner radius (*R*), and the thickness of the spherical head (*T*_*h*_).

**Figure 2:**
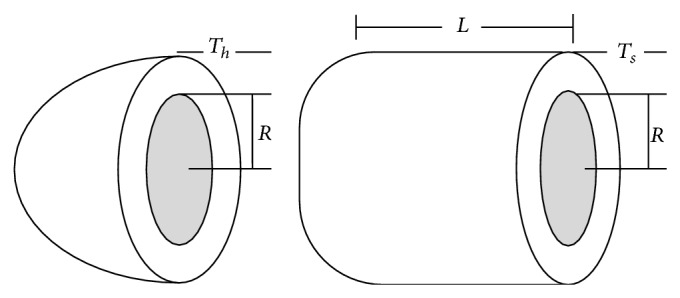
Schematic of the pressure vessel design problem [1].

The variables *T*_*s*_ and *T*_*h*_ are discrete values which are integer multiples of 0.0625 inches.  Figure 2 shows the cylindrical pressure vessel capped at both ends by hemispherical heads.

Assuming *x*_1_ = *T*_*s*_, *x*_2_ = *T*_*h*_, *x*_3_ = *R*, and *x*_4_ = *L* as the design variables, the optimization problem can be mathematically expressed as follows:

(4) Minimize f(x→)=0.6224x1x3x4+1.7781x2x32        +3.1611x12x4+19.8621x12x3,Subject to g1x→=0.0193x3−x1≤0,      g2(x→)=0.00954x3−x2≤0,      g3(x→)=x4−240≤0,      g4(x→)=750×1728−πx32x4          −43πx33≤0.

The simple bounds of the problem are *x*_1_, *x*_2_ ∈ [1 × 0.0625, 99 × 0.0625] and *x*_3_, *x*_4_ ∈ [10.0, 240.0]. Unlike the usual limit of 200 in considered in literatures, the upper bound of design variable* L* was increased to 240 in to expand the search space.

Optimization results are presented in Table 5. GDE3 produced a design result with a value of 6083.773 within 400 iterations (i.e., 10,000 evaluations).  Table 6 compares the optimal design results produced by GDE3 with those reported in [1, 17, 20, 21, 24, 32]. Further, a statistical evaluation of 100 independent runs of the GDE3 metaheuristic is tabulated in Table 7 considering the best, worst, average, and the standard deviation (std. dev.) of the obtained solutions. The ratio between the optimized costs corresponding to worst and best designs is 1.00229. The best design result was produced by the Firefly algorithm. GDE3 metaheuristic produced the least performance compared to the other algorithms.

**Example 3 (speed reducer design optimization problem).**

**Figure 3:**
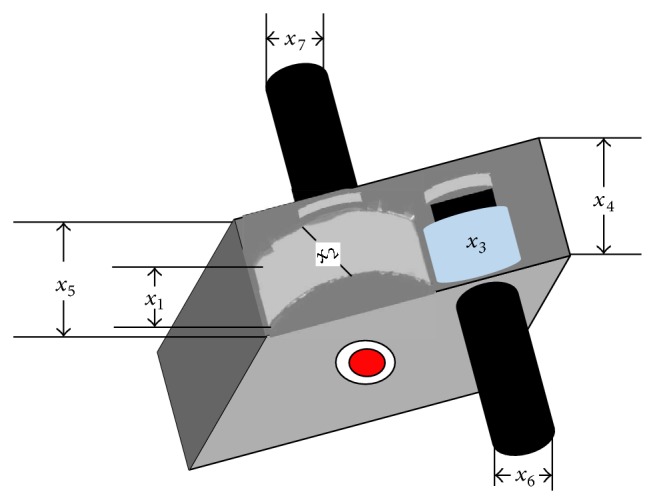
Schematic of the speed reducer design problem [25].

The speed reducer design problem [25] is designed to minimize the weight of the speed reducer subjecting it to some constraints such as shaft stresses, surface stress, gear teeth bending stress, and shafts crosswise deflections. The width of the gear face *x*_1_, teeth module *x*_2_, number of pinion teeth *x*_3_, first shaft length between bearings *x*_4_, second shaft length between bearings *x*_5_, the diameter of the first shaft *x*_6_, and diameter of the second shaft are the design variables of the optimization problem.  Figure 3 shows the schematic of the speed reducer.

The mathematical expression for the speed reducer problem is as follows:

(5) Minimize f(x→)=0.7854x1x22          ·3.3333x32+14.9334x3         −43.0934x32         −1.508x1(x62+x72)         +7.4777(x63+x73)        +0.7854x4x62+x5x72,Subject to g1x→=27x1x22x3−1≤0,       g2x→=397.5x1x22x32−1≤0,       g3(x→)=1.93x43x2x3x64−1≤0,         g4(x→)=1.93x53x2x3x74−1≤0,         g5(x→)=1.0110x63             ·745.0x4x2x32+16.9×106          −1≤0,         g6(x→)=1.085x73           ·745.0x5x2x32+157.5×106          −1≤0,          g7(x→)=x2x340−1≤0,         g8(x→)=5x2x1−1≤0,         g9(x→)=x112x2−1≤0,        g10(x→)=1.5x6+1.9x4−1≤0,          g11(x→)=1.1x7+1.9x5−1≤0.

The simple bounds of the problem are *x*_1_ ∈ [2.6, 3.6], *x*_2_ ∈ [0.7, 0.8],  *x*_3_ ∈ [17, 28], *x*_4_ ∈ [7.3, 8.3],  *x*_5_ ∈ [7.8, 8.3],  *x*_6_ ∈ [2.9, 3.0], and *x*_7_ ∈ [5.0, 5.5].

The optimum design of the speed reducer is executed using GDE3 metaheuristic, and the best solution is found as *x* ^*^ = {*x*_1_,  *x*_2_,  *x*_3_,  *x*_4_,  *x*_5_,  *x*_6_,  *x*_7_} = {3.50000000047883, 0.7, 17.0, 7.3, 7.8, 3.35021466645262, 5.2866832298256} which yields an objective function value of $f(\vec{x})$ = 2996.34816529042 as seen in Table 8.

The results obtained by GDE3 are presented in Table 8. GDE3 found the global optimum requiring 400 iterations per optimization run.  Table 9 provides a comparison of this solution with the results of simple constrained particle swarm optimization. It is apparent from the table that GDE3 metaheuristic finds a competitive solution using only 10,000 objective function evaluations, which is considerably lesser than those of other approaches. Further, a statistical evaluation of 100 independent runs of the GDE3 metaheuristic is tabulated in Table 10 considering the best, worst, average, and the standard deviation (std. dev.) of the obtained solutions. The ratio between the optimized costs corresponding to best and worst designs is 1.0000003. Remarkably, GDE3 produced the overall best design result with a value of 2996.3481653.

**Example 4 (tension/compression spring design optimization problem).**

**Figure 4:**
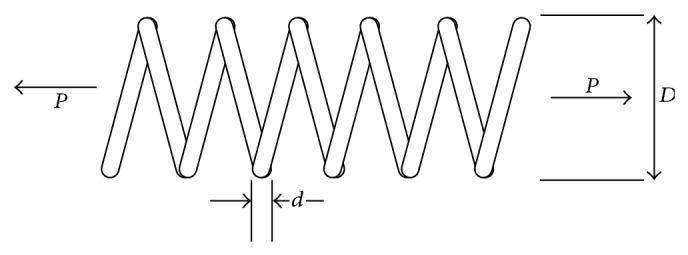
Schematic of the tension/compression spring design problem.

The tension/compression spring problem is designed to minimize the weight of the spring subjecting it to some constraints such as shear stress, minimum deflection, outside diameter limits, and surge frequency. The design variables are the number of active coils *P*, the diameter of the mean coil *D*, and the diameter of the wire *d*.  Figure 4 shows the tension/compression spring design.

Assuming *x*_1_ = *d*, *x*_2_ = *D*, and *x*_3_ = *P*, as the design variables, the tension/compression spring design problem can be expressed as follows:

(6) Minimize fx→=x3+2x2x12,Subject to g1(x→)=1−x23x371,785x14≤0,      g2(x→)=4x22−x1x212,566(x2x13−x14)          +15,108x12−1≤0,        g3(x→)=1−140.45x1x22x3≤0,        g4(x→)=x2+x11.5−1≤0.

The simple bounds of the problem are *x*_1_ ∈ [0.05, 2.0], *x*_2_ ∈ [0.25, 1.3], and *x*_3_ ∈ [2.0, 15.0].

The optimum design of the tension/compression spring is carried out using GDE3 metaheuristic, and the best solution is found as *x* ^*^ = {*x*_1_, *x*_2_, *x*_3_} = {0.0517955276224998, 0.359283196922392, 11.1405163630287} which yields an objective function value of $f(\vec{x})$ = 0.0126658360085857 as seen in Table 11.

The results obtained by GDE3 are presented in Table 11. GDE3 found the global optimum requiring 400 iterations per optimization run.  Table 12 provides a comparison of this solution with the results of simple constrained particle swarm optimization. It is apparent from the table that GDE3 metaheuristic finds a competitive solution using only 10,000 objective function evaluations, which is considerably lesser than those of other approaches. Further, a statistical evaluation of 100 independent runs of the GDE3 metaheuristic is tabulated in Table 13 considering the best, worst, average, and the standard deviation (std. dev.) of the obtained solutions. The ratio between the optimized costs corresponding to worst and best designs is 1.000107.

## 4. Conclusion

In the present study, the GDE3 algorithm is used as a simple and efficient optimization technique for handling engineering optimization problems. The GDE3 algorithm also uses a very simple mechanism to deal with constrained functions and results generated by the algorithm indicate that such mechanism, despite its simplicity, is effective in practice. From this study, performance evaluation of the GDE3 algorithm through benchmark design optimization examples reveals the efficiency of this technique in solving practical optimization problems. Although in the present study the algorithm is utilized only for solving engineering design optimization problems, GDE3 algorithm can easily be employed for solving other types of optimization problems as well.

## Figures and Tables

**Algorithm 1:**
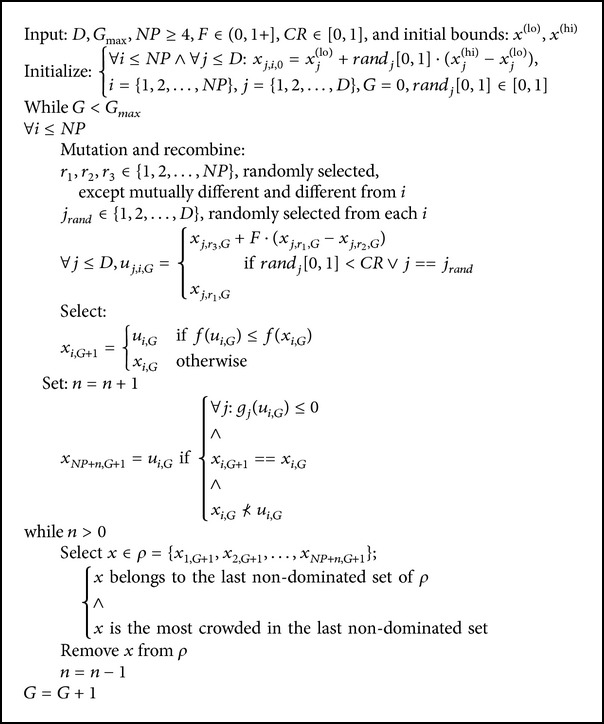
The GDE3 algorithm [29].

**Table 1:** Values of parameters involved in the formulation of the welded beam problem [1].

Constant item	Description	Values
*C*_1_	The welded material	0.10471 ($/in3)
*C*_2_	The bar stock	0.04811 ($/in3)
*τ*_max⁡_	Shear stress of the welded material	13600 (psi)
*σ*_max⁡_	Normal stress of the bar material	30000 (psi)
*δ*_max⁡_	Bar end deflection	0.25 (inch)
*E*	Young's modulus of bar stock	30 × 10^6^ (psi)
*G*	Shear modulus of bar stock	12 × 10^6^ (psi)
*P*	Loading condition	6000 (lb)
*L*	Beam's projection length	14 (inch)

**Table 2:** GDE3 solution vector for welded beam.

Best solution	*x*_1_	*x*_2_	*x*_3_	*x*_4_
	0.20572840999876	3.47072911158159	9.03661683005891	0.20572540074781
	$g_{1}(\vec{x})$	$g_{2}(\vec{x})$	$g_{3}(\vec{x})$	$g_{4}(\vec{x})$
	−0.66062798472194	0.665171394633944	3.00925094998*E* − 06	−3.43299575416113
	$g_{5}(\vec{x})$	$g_{6}(\vec{x})$	$g_{7}(\vec{x})$	$f(\vec{x})$
	−0.08072840999876	−0.235539990649711	0.373971078704926	1.72484969509211

**Table 3:** Welded beam problem: comparison of GDE3 results with other optimization methods.

Researcher	Metaheuristic	*x*_1_	*x*_2_	*x*_3_	*x*_4_	*f*(*x*)	NE
[10]	Genetic algorithm	0.2489	6.1730	8.1789	0.2533	2.4331	320,080
[11]	Genetic algorithm	0.2489	6.1097	8.2484	0.2485	2.4000	6,273
[12]	Social behavioral model	0.2407	6.4851	8.2399	0.2497	2.4426	19,259
[13]	Society and civilization algorithm	0.2444	6.2380	8.2886	0.2446	2.3854	33,095
[14]	Genetic algorithm	0.2443	6.2117	8.3015	0.2443	2.3816	320,000
[15]	Particle swarm optimization	0.2444	6.2175	8.2915	0.2444	2.3810	30,000
[16]	Harmonic search	0.2442	6.2231	8.2915	0.2443	2.3810	110,000
[17]	Simulated annealing—direct search	0.2444	6.2158	8.2939	0.2444	2.3811	56,243
[18]	Simulated annealing—genetic algorithm	0.2231	1.5815	12.8468	0.2245	2.2500	26,466
[19]	Artificial Immune System—genetic algorithm	0.2444	6.2183	8.2912	0.2444	2.3812	320,000
[20]	Harmonic search	0.2057	3.4705	9.0366	0.2057	1.7248	200,000
[21]	Simple constrained particle swarm optimizer	0.2057	3.4705	9.0366	0.2057	1.7249	24,000
[22]	Harmonic search—sequential quadratic programming	0.2057	3.4706	9.0368	0.2057	1.7248	90,000
[23]	Differential evolution	0.2444	6.2175	8.2915	0.2444	2.3810	24,000
[8]	Evolutionary algorithm	0.2443	6.2201	8.2940	0.2444	2.3816	28,897
[1]	Firefly algorithm	0.2015	3.5620	9.0414	0.2057	1.7312	50,000
[24]	Simple optimization	0.2057	3.4705	9.0366	0.2057	1.7246	10,000
Present study	Generalized differential evolution 3	**0.2057**	**3.4707**	**9.0366**	**0.2057**	**1.724849**	**10,000**

**Table 4:** Statistical results of the GDE3 optimization.

Best	Average	Worst	Std. dev.	Number of iterations
1.724849	1.725023	1.725569	0.0001018	400

**Table 5:** GDE3 Solution vector for pressure vessel.

Best Solution	*x*_1_	*x*_2_	*x*_3_	*x*_4_
	0.74395291436715	0.36774755668330	38.5288195380221	239.37719314082
	$g_{1}(\vec{x})$	$g_{2}(\vec{x})$	$g_{3}(\vec{x})$	$g_{4}(\vec{x})$
	−0.00034669728332	−0.00018261829057	−0.62280685917400	−42.436889517499
	$f(\vec{x})$			
	6083.77328355025			

**Table 6:** Pressure vessel problem: comparison of GDE3 results with optimization methods.

Researcher	Metaheuristic	*x*_1_	*x*_2_	*x*_3_	*x*_4_	*f*(*x*)
[17]	Simulated annealing—direct search	0.7683	0.3797	39.8096	207.2250	5868.76
[32]	Particle swarm optimization—genetic algorithm	0.7500	0.3750	38.8601	221.3654	5850.383
[20]	Harmonic search	0.7500	0.3750	38.8600	221.3600	5849.7
[21]	Simple constrained particle swarm optimizer	0.8125	0.4375	42.0980	176.6360	6.059.714
[1]	Firefly algorithm	0.7500	0.3750	38.8600	221.3600	5850.3
[24]	Simple optimization	1.1250	0.6250	58.2901	43.6927	7199.35
Present study	Generalized differential evolution 3	0.74391	0.36774	38.5288	239.377	6083.773

**Table 7:** Statistical results of the GDE3 optimization.

Best	Average	Worst	Std. dev.	Number of iterations
6083.773	6092.318	6097.725	40.32205	400

**Table 8:** GDE3 Solution vector for speed reducer.

Best Solution	*x*_1_	*x*_2_	*x*_3_	*x*_4_
	3.5000000004788	0.7000000000000	17.0000000000000	7.3000000000000
	*x*_5_	*x*_6_	*x*_7_	$g_{1}(\vec{x})$
	7.8000000000000	3.3502146664526	5.2866832298256	−0.07391528052456
	$g_{2}(\vec{x})$	$g_{3}(\vec{x})$	$g_{4}(\vec{x})$	$g_{5}(\vec{x})$
	−0.197998527251663	−0.499172248315386	−0.9014716976203	−3.1892233298*E* − 10
	$g_{6}(\vec{x})$	$g_{7}(\vec{x})$	$g_{8}(\vec{x})$	$g_{9}(\vec{x})$
	−3.8408276559*E* − 11	−0.7025	−1.3680001575*E* − 10	−0.5833333332763
	$g_{10}(\vec{x})$	$g_{11}(\vec{x})$	$f(\vec{x})$
	−0.0513257534686439	−0.0108523650245949	2996.34816529042

**Table 9:** Speed reducer problem: comparison of generalized differential evolution 3 results with simple constrained particle swarm optimization.

Solution	Simple constrained particle swarm optimization [21]	Generalized differential evolution (Present study)
*x*_1_	3.5000	3.5000
*x*_2_	0.7000	0.7000
*x*_3_	17.0000	17.0000
*x*_4_	7.3000	7.3000
*x*_5_	7.8000	7.8000
*x*_6_	3.350214	3.3502146
*x*_7_	5.286683	5.2866832
$f(\vec{x})$	2996.348165	2996.3481653

**Table 10:** Statistical results of the GDE3 optimization.

Best	Average	Worst	Std. dev.	Number of iterations
2996.3481653	2996.3483815	2996.3491534	0.0000021	400

**Table 11:** GDE3 Solution vector for tension/compression spring.

Best Solution	*x*_1_	*x*_2_	*x*_3_	$g_{1}(\vec{x})$
	0.0517955276224998	0.359283196922392	11.1405163630287	−3.0601282864*E* − 05
	$g_{2}(\vec{x})$	$g_{3}(\vec{x})$	$g_{4}(\vec{x})$	$f(\vec{x})$
	−0.133636716257444	−4.05865278285946	−0.725947516970072	0.01266583600858

**Table 12:** Tension/compression spring problem: comparison of GDE3 results with simple constrained particle swarm optimization.

Solution	Simple constrained particle swarm optimization [21]	Generalized differential evolution 3
*x*_1_	0.051583	0.0517955
*x*_2_	0.354190	0.3592831
*x*_3_	11.438675	11.140516
$f(\vec{x})$	0.012665	0.012665836

**Table 13:** Statistical results of the GDE3 optimization.

Best	Average	Worst	Std. dev.	Number of iterations
0.012665836	0.012666648	0.012667194	3.97815*E* − 07	400
